# Effects of syntactic context on eye movements during reading

**DOI:** 10.2478/v10053-008-0078-0

**Published:** 2010-10-29

**Authors:** Lynn Huestegge, Diana Bocianski

**Affiliations:** Institute of Psychology, RWTH Aachen University, Germany

**Keywords:** reading, eye movements, syntax, context, global effects, syntactic priming

## Abstract

Previous research has demonstrated that properties of a currently fixated word and of adjacent words influence eye movement control in reading. In contrast to such local effects, little is known about the global effects on eye movement control, for example global adjustments caused by processing difficulty of previous sentences. In the present study, participants read text passages in which voice (active vs. passive) and sentence structure (embedded vs. non-embedded) were manipulated. These passages were followed by identical target sentences. The results revealed effects of previous sentence structure on gaze durations in the target sentence, implying that syntactic properties of previously read sentences may lead to a global adjustment of eye movement control.

## INTRODUCTION

Since the earliest days of psychology, the explanation of spatial and temporal variability of eye fixations has been a constant challenge. In response to this challenge, experimental studies revealed multiple sources of influence on eye movement control, which can be placed on a continuum ranging from local to global (e.g., Pynte & Kennedy, 2006). *Local effects* subsume any influence of properties of the currently fixated information, including input within the current perceptual span, that is, the region around a current fixation within which information can be extracted. In contrast, *global effects* refer to a comparatively long-lasting adjustment of oculomotor patterns based on task demands, the subject’s overall goals, or general properties of previously processed information outside the current perceptual span. Although substantial effort has been put into the exploration (and modelling) of local influences (see Rayner, 2009), global factors have been largely neglected. The present study aims at specifying global effects in the domain of reading. More specifically, we ask whether reading of a particular syntactic structure of previous text passages may lead to a global adjustment of eye movement control during the reading of a following identical target sentence.

A very influential account regarding the local control of eye movements in reading was proposed by Just and Carpenter (1980) , mainly stating that information processing is spatially and temporally tightly coupled to the currently fixated word. Indeed, it was shown that word length and frequency directly influence oculomotor processing of the respective word (see Rayner, 1998). Additionally, prolonged fixation durations were also demonstrated for words that are unpredictable, semantically implausible, or violate semantic expectancies (e.g., Balota, Pollatsek, & Rayner, 1985; Ehrlich & Rayner, 1981; Morris, 1994; Rayner, Warren, Juhasz, & Liversedge, 2004). Even though in these examples the preceding context outside the current perceptual span may play a crucial role, such findings still qualify as local effects since the relevant source of influence is, for example, the unexpectedness of a currently fixated word.

However, further evidence suggested that the coupling between the current fixation position and currently processed information could be much looser. More specifically, properties of words *n*-1 and *n*+1 may also affect eye movement patterns on word n. For example, *the spillover effect* is the tendency of the eye to remain longer on a word when the previous was a low frequency word compared to a high frequency word (Rayner & Duffy, 1986). Furthermore, a recent regression analysis of corpus data suggested that several words within the perceptual span might be processed in parallel on different perceptual and cognitive levels (Kliegl, Nuthmann, & Engbert, 2006; but see also Reichle, Liversedge, Rayner, & Pollatsek, 2009). In addition to such local influences on the level of word recognition, there is also evidence for syntactic, semantic, and pragmatic influences on oculomotor control on the level of sentence comprehension (higher-level local effects, see Rayner, 1998, for a review). For example, syntactic and semantic ambiguity or anomaly is known to affect fixation times and/or the occurrence of regressions (i.e., saccades back to previously inspected text) for disambiguation purposes (for a recent review, see Rayner, 2009). Similarly, syntactic complexity (e.g., when a verb’s object appears not adjacent to the verb but at the end of a sentence) is known to play a role in eye guidance (see Clifton, Staub, & Rayner, 2007, for a review). Again, even though information outside the current perceptual span has to be integrated for sentence processing, these findings qualify as local effects, since currently fixated information (e.g., a syntactically ambiguous word) is the primary source of influence on eye movement control.

In contrast to the study of local influences, only few studies addressed the issue of global effects on eye movements in reading. For example, some studies reported effects of interindividual differences in working memory span (Kennison & Clifton, 1995), differences in reading skill (Huestegge, Radach, Corbic, & Huestegge, 2009; Rayner & Juhasz, 2004), or different reading strategies and/or intentions (Heller, 1982; Hyönä, Lorch, & Kaakinen, 2002; O’Regan & Levy-Schoen, 1987; Radach, Huestegge, & Reilly, 2008). While these findings show that readers are able to adjust globally their eye movement patterns, the sources of these effects are located in the reader and not in the text. Studies that addressed global manipulations on text-level are even rarer. For example, a study by Vauras, Hyönä, and Niemi (1992) reported effects of text coherence. They found significantly more regressions towards incoherent text segments and worse recall performance for incoherent text (i.e., text in which a change of sentence order induced incoherence). Other studies found that the language in which a text was written (e.g., Kennedy & Pynte, 2005, who found that French text is generally processed more slowly than English text) or the overall text difficulty (Rayner, Chase, Slattery, & Ashby, 2006) may affect overall text processing speed. However, it remained unclear to what extent these findings really represent global effects, since the locus of the manipulation and the locus of measurement coincide, and any changes in the eye movement record can be attributed principally to local effects.

More direct evidence for global effects of text surroundings on current eye movement control was reported by Radach et al. (2008). They demonstrated that the format in which a text was presented (single sentences vs. the same sentences embedded in text passages) significantly affected eye movements during reading. Furthermore, Pynte and Kennedy (2006) found that an increase of the average word length of words n-4 to n-10 went hand in hand with an increase of the mean number of fixations on word n. They interpreted this finding in terms of a global fine-tuning mechanism in response to previously experienced text parameters. However, these findings were based on a quasi-experimental design, and further experimental evidence is needed to support this claim of text-based global tuning mechanisms in oculomotor control.

A separate line of evidence supporting the idea of global tuning mechanisms comes from syntactic priming studies. Originally, syntactic priming referred to the tendency to repeat syntactic structure across utterances, but similar effects were shown in the domain of language comprehension (Branigan, 2007; Branigan, Pickering, & McLean, 2005; Frazier, Munn, & Clifton, 2000; Frazier, Taft, Roeper, Clifton, & Ehrlich, 1984). For example, reading times for a phrase were reported to be shorter when the previous sentence shared its syntactic structure (Staub & Clifton, 2006). However, these effects hinged on the presence of cues in the sentence that were informative regarding its syntactic similarity to the previous sentence, for example by repeating specific words or commas in the prime and target sentence. Thus, it remains an open question to what extent syntactic priming might play a role during the reading of natural text passages (see Clifton et al., 2007).

**Figure 1.**
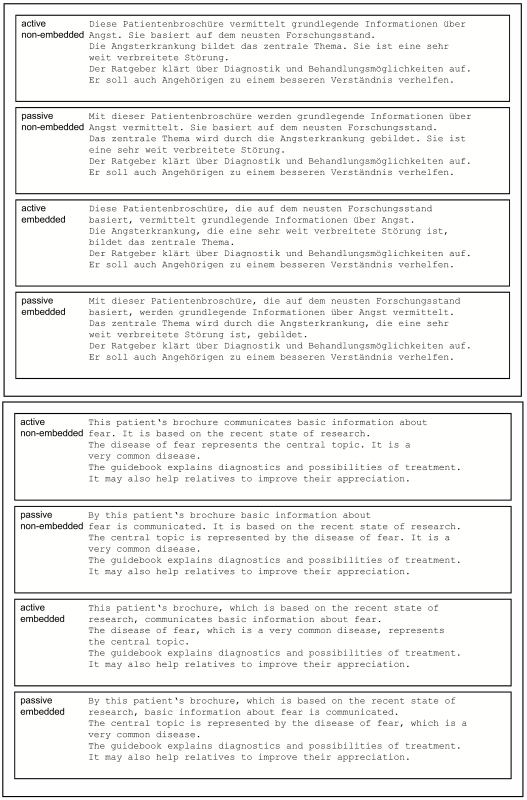
Example of the manipulated text passages. The figure shows active non-embedded, passive non-embedded, active embedded, and passive embedded sentence structures in the first four lines. Lines 5 and 6 remained unchanged. Each subject read a continuous text consisting of four blocks with 14 passages each. English glosses (lower panel) are translated from the original German text passages (upper panel).

Taken together, up to now, there has been no experimental evidence for global tuning mechanisms during reading of natural text passages that result from differences within the text material. In the present experiment, we address this issue by presenting participants with semantically identical text passages, which include either active or passive voice constructions, and embedded or non-embedded sentence structures (see Figure 1). We reasoned that these syntactic manipulations allow us to vary the overall difficulty of text processing without changing much of the physical visual input, including overall word length and frequency. Crucially, these passages were followed by identical target sentences, on which a global adjustment of eye movement control may occur. The comparatively subtle manipulations of syntax structure were chosen to minimize the likelihood of local effects to occur. For example, a pronounced difference in semantic processing ease (e.g., by varying the number of low frequency words or foreign words) between the context and the target sentence would probably yield local surprise effects during reading of the target sentence.

As a by-product, the present design also allows us to assess to what degree passive (vs. active) voice and embedded (vs. non-embedded) sentence structures hamper reading performance, a claim that has often been raised by highly influential guidelines of writing style (e.g., APA, 2001; Strunk & White, 1918/1999). Previous research suggested an advantage of active over passive voice on recall parameters (Coleman, 1965), and linguistic theories about text comprehensibility often assume that active voice and simple sentence structures should enhance processing ease (see e.g., Groeben & Christmann, 1989, for an example in German). However, these assumptions have not been thoroughly backed by empirical studies.

## Method

### Participants

Thirty-two native German speakers (20 female and 12 male students, aged 21-28 years) with normal or corrected-to-normal vision took part in the experiment.

### Apparatus

Participants were seated with a chin rest 60 cm in front of a 21” monitor (1024 × 768 pixels resolution) running at 120 Hz. Eye movements were recorded using an Eyelink II system (SR Research) with a temporal resolution of 500 Hz and a spatial resolution of < 0.022°.

### Material

The text material was constructed around a German brochure on fear. The final text consisted of 56 semantically consecutive passages, each containing six lines of text (60-68 characters per line). The first four lines were manipulated syntactically according to a 2 × 2 design, whereas the last two lines always remained unchanged. The first four lines included either active vs. passive voice sentences and embedded vs. non-embedded structures, while words and semantics remained unchanged as much as possible. The first two lines either consisted of (a) two sentences, both in active voice (*active non-embedded*), or (b) of two sentences, with the first in passive voice and the second unchanged (*passive non-embedded*), or (c) one sentence in active voice, with the second sentence embedded as a relative clause (*active embedded*), or (d) one sentence in passive voice, with the second sentence embedded as a relative clause (*passive embedded*). The same manipulations were implemented within lines 3 and 4 to provide a context large enough to allow an impact on further reading. As a result of the manipulations, mean word count and mean number of characters on lines 1-4 varied across conditions; (a) 30.9/217.1, (b) 31.0/234.1, (c) 30.6/219.0, (d) 32.7/235.1 words/characters, respectively. Line 5 consisted of the unchanged target sentence. Line 6 contained an additional unchanged sentence (see Figure 1). All versions of the passages were presented to three lecturers in linguistics who confirmed that all variants were natural and plausible.

### Procedure and design

Participants were instructed to read the text silently for comprehension. They responded orally to a comprehension question as accurately as possible after each passage (e.g., “What can be very expensive?” − “Good treatment by a competent doctor”). Of all the questions, 25% of them targeted material in the target sentence. Examples of correct answers were given during practice trials. The answers to the questions were written down by the experimenter and afterwards used to compute a comprehension score for each subject by counting every correctly reproduced adjective and substantive (see Huestegge et al., 2009; Radach et al., 2008, for successful implementations of this procedure). Note that this specific scoring procedure usually leads to substantially lower comprehension scores than do procedures involving only a semantically correct rephrasing of the previous passage. Each passage (always preceded by a 9-point calibration) was presented when participants pressed the space bar of the keyboard. When they finished reading, they pressed the space bar again, and the question was displayed.

Each subject read 56 passages, which were divided into four blocks of 14 passages that were each presented in one syntactic condition. For example, one subject started with 14 active/embedded passages, continued with 14 passive/non-embedded passages, etc. The condition sequence was counterbalanced according to a Latin square design, resulting in four groups of participants. The block design was chosen to maximize the chance for global tuning effects to occur, since global oculomotor routines may establish only in the course of several text passages posing a specific processing demand. The experiment lasted about 90 min.

Text passages were presented in monospaced black font on a white background. Each letter comprised a visual angle of about 1/3°.

A 2 × 2 repeated measurement ANOVA based on subject means with the independent variables voice (active vs. passive) and embeddedness (embedded vs. non-embedded) was conducted (significance level = .05). The results will focus primarily on word-based reading measures, since oculomotor control in reading is known to be word-based (Rayner, 1998), and any global tuning mechanism is thus likely to occur in word-based parameters in the target sentence, such as gaze durations (defined as the sum of durations of all fixations on a word until the word is left for the first time). We additionally report item-based analyses (*F*_2_) except for the analysis of comprehension scores, which represent aggregate measures across all passages within a block. Due to the overall large amount of dependent variables (see Table 1 and 2), we will provide a somewhat condensed overview of the most important results to maintain readability.

**Table 1.** Oculomotor Parameters on Text Line 5 (target sentence): Means and Standard Errors of Eye Movement Parameters on the Target Sentence (line 5) of the Passages as a Function of the Previously Read Sentence Structure (active vs. passive and embedded vs. non-embedded).

		Number of fixations on sentence (*N*)		Progressive saccade amplitude (letter units)		Regression rate (%)		Total sentence reading time (s)		Number of fixations per word		Gaze duration per word (ms)		Total reading time per word(ms)	
		Mean	*SE*	Mean	*SE*	Mean	*SE*	Mean	*SE*	Mean	*SE*	Mean	*SE*	Mean	*SE*
active	non-embedded	15,4	0,9	6,9	0,3	16,5	0,8	3,75	0,2	2,13	0,11	273	7,9	439	22,8
	embedded	14,7	0,7	6,8	0,3	15,4	1,0	3,54	0,2	2,07	0,09	282	9,2	424	17,6
passive	non-embedded	14,9	0,9	6,9	0,2	16,6	0,9	3,61	0,2	2,07	0,11	271	6,6	426	24,0
	embedded	15,2	0,8	6,8	0,3	16,5	0,9	3,68	0,2	2,17	0,10	285	9,7	444	20,5

**Table 2.** Oculomotor Parameters on the Syntactically Manipulated Text Lines 1 to 4: Means and Standard Errors of Eye Movement Parameters on the First Four Lines of the Passages as a Function of Sentence Structure (active vs. passive and embedded vs. non-embedded).

		Number of fixations on lines 1-4 (*N*)		Progressive saccade amplitude (letter units)		Regression rate (%)		Overall reading time on lines 1-4 (s)		Number of fixations per word		Gaze duration per word (ms)		Total reading time per word(ms)	
		Mean	*SE*	Mean	*SE*	Mean	*SE*	Mean	*SE*	Mean	*SE*	Mean	*SE*	Mean	*SE*
active	non-embedded	58,6	3,2	6,7	0,2	23,8	0,8	15,63	0,80	2,63	0,14	311	10,7	530	25,8
	embedded	59,5	3,3	6,8	0,2	23,9	0,8	15,71	0,82	2,63	0,12	309	7,5	530	24,8
passive	non-embedded	56,9	3,2	6,8	0,2	23,5	0,9	16,17	0,84	2,46	0,11	308	8,1	510	21,7
	embedded	62,8	3,4	6,6	0,2	23,5	0,8	16,50	0,87	2,79	0,14	329	9,3	558	25,4

## Results

### Overall text comprehension

Comprehension scores were lower for embedded, *M* = 41.03% correct, *SE* = 2.07, compared to non-embedded structures, *M* = 55.85% correct, *SE* = 2.72, *F*(1, 31) = 37.1, *p *< .001. This is clear evidence for increased processing difficulty while reading embedded structures. There was no significant main effect of voice, active: *M* = 48.76%, *SE* = 2.55, passive: *M* = 48.13%, *SE* = 2.23, *F *< 1; and no significant interaction, *F*(1, 31) = 1.7, *p *= .206.

### Global effects on the identical target sentence (line 5)

Table 1 presents an overview of the relevant eye movement parameters on the target sentence. Prior to the computation of word-based parameters, the first and last word of the line 5 were excluded to rule out effects of sentence wrap-up processing. Most importantly, mean gaze durations (sum of all fixations until the word is left for the first time) on words in the target sentence were significantly greater following embedded sentence structures compared to non-embedded structures, *F*_1_(1, 31) = 4.2, *p *= .049; *F*_2_(1, 55) = 5.5, *p *= .023. There was neither a significant main effect of voice, nor an interaction (all *Fs* < 1). This finding is clear evidence for a change in oculomotor patterns resulting from the previously experienced sentence structure.

Interestingly, there were no significant main effects on mean total reading times per word, which include all fixations on the word (all *Fs* < 1), probably partly due to the overall high standard errors compared to those for gaze durations (see Table 1). However, a significant interaction of voice and embeddedness was present, *F*_1_(1, 31) = 4.4, *p *= .044; *F*_2_(1, 55) = 3.4, *p *= .070. A closer inspection of the data revealed that in the passive conditions, the mean total reading time per word was 18 ms greater for embedded as compared to non-embedded structures. Thus, the effect of embeddedness on gaze durations was also reflected in total reading times per word, but only in passive conditions. Surprisingly, in active conditions, total reading times per word tended to be shorter (15 ms) after reading embedded as compared to non-embedded structures.

Initial landing positions (overall M/*SE* for four-, five-, six-, seven-, and eight-letter words amounted to 2.25/0.09, 2.42/0.10, 2.89/0.12, 3.16/0.24, 3.47/0.28, respectively) and word skipping rate (overall *M* = 19.15, *SE* = 1.16) while reading the target sentence did not significantly differ as a function of voice or embeddedness, all *Fs* < 1. The mean number of fixations on the whole target sentence (*M* = 15) and the mean total sentence reading time (including all fixations on the sentence) were also not significantly affected by the syntactic structure of the previous text, all *Fs* < 2. Note, however, that there was a tendency towards more fixations and increased sentence reading times for embedded vs. non-embedded structures in passive conditions, while this pattern tended to be reversed for active conditions (similar to the total reading times per word, see Table 1). There were no significant effects on mean fixation durations (overall *M* = 213, *SE* = 3.82), regression rate (relative occurrence of saccades back to previously inspected text), and the mean amplitude of saccades in reading direction. Additional analyses on line 6 revealed no significant effects at all, indicating decay of global tuning mechanisms two sentences after the experimental manipulation.

### Eye movements on the manipulated sentence structures (lines 1 to 4)

Eye movements on the manipulated sentence structures (lines 1 to 4) were additionally measured to see whether the processing difficulty for embedded structures reported above is also reflected in the eye movement record. Table 2 presents an overview of the temporal and spatial parameters as a function of experimental conditions. The mean number of fixations on lines 1 to 4 was significantly smaller for non-embedded as compared to embedded sentence structures in the item analysis, although this effect was only marginally significant in the subject-based analysis, *F*_1_(1, 31) = 3.8, *p *= .062; *F*_2_(1, 55) = 16.9, *p *< .001. This may be interpreted as a reflection of increased processing difficulty for embedded structures. There was neither a significant main effect of voice (all *Fs* < 1), nor a significant interaction, *F*_1_(1, 31) = 2.7, *p *= .110, *F*_2_ < 1.

Interestingly, the overall reading time for all sentences on lines 1 to 4 was not affected by embeddedness (*F *< 1), but it was significantly prolonged for passive compared to active sentences, *F*_1_(1, 31) = 5.7, *p *= .023; *F*_2_(1, 55) = 2.0, *p *= .158. There was no significant interaction (all *Fs* < 1).

The regression rate (percentage of saccades directed against reading direction) did not change as a function of embeddedness or voice, and there was no interaction (all *Fs* < 1). The length of saccade amplitudes in reading direction also did not significantly change as a function of the conditions.

In addition to these sentence-related measures, we computed mean oculomotor parameters for words that received at least one fixation. We discarded data on the first and last words of each line to exclude effects related to return sweeps. The mean number of fixations per word was greater for embedded as compared to non-embedded sentence structures, *F*_1_(1, 31) = 5.6, *p *= .024; *F*_2_(1, 55) = 8.5, *p *= .005, reflecting the corresponding sentence-related measure reported above. However, the significant interaction of voice and embeddedness, *F*_1_(1, 31) = 5.5, *p *= .026; *F*_2_(1, 55) = 4.7, *p *= .035, indicates that this effect was only present in passive sentence environments (see Table 2). There was no main effect of voice (all *Fs* < 1). Gaze durations and total reading times per word did not significantly differ as a function of the conditions. However, it is noted that for passive voice conditions, these parameters tended towards greater values for embedded (vs. non-embedded) structures (about 20 ms for total reading times per word), thus resembling the results regarding the mean number of fixations per word: interaction of voice and embeddedness for total reading times per word: *F*_1_(1, 31) = 3.0, *p *= .095; *F*_2_(1, 55) = 4.8, *p *= .034. Mean initial landing positions were computed separately for four-, five-, six-, seven-, and eight-letter words, but no significant effects were found within each word length, all *Fs* < 1.

## Discussion

The present experiment was conducted to determine whether reading of syntactically varied prose passages might lead to a global adjustment of eye movement control during reading of subsequent identical target sentences. Overall, the comprehension scores indicated that processing difficulty was greater for reading embedded as compared to non-embedded sentence structures. The eye movement record partly reflected this processing difficulty: Embedded structures yielded an increased number of fixations, and a tendency for increased gaze durations and total reading times per word (in passive conditions). This difficulty for processing embedded sentences may result from a higher working memory load: In centre-embedded structures, the relative clause has to be processed while the information of the preceding part needs to be stored in working memory for subsequent integration purposes (see Caplan, Alpert, & Waters, 1998; Gibson, 1998).

Most importantly, reading embedded structures increased overall gaze durations on subsequent identical target sentences. In line with the quasi-experimental study of Pynte and Kennedy (2006) , this can be interpreted as experimental evidence for a global adjustment of word-based eye movement control, according to which the processing difficulty regarding the first four lines led to globally adjusted gaze durations on the subsequent sentence. Since gaze durations reflect first-pass reading (i.e., fixations from the time the gaze first enters a word until it moves outside the word for the first time), it is safe to say that the initial processing of words was prolonged after reading embedded structures.

However, the results are more ambivalent with respect to parameters that additionally involve second-pass reading (i.e., when the reader fixates a word after it has been fixated and exited for the first time, sometimes-referred to as late measures). These parameters include total reading times per word, the total number of fixations per word, and total sentence reading times. While the effect of embeddedness on gaze durations seems to carry over to these measures in passive conditions (see Table 1), the overall pattern tends to be reversed in active conditions, as indicated by the significant interaction in total reading times per word. One potential reason for this deviating pattern in active conditions might be the overall greater variability of these late measures (see Table 1), which mirror a great variety of processing from lexical to sentence level (see Rayner, 1998). However, the consistency of this pattern among all of these late measures and a corresponding tendency in the regression rates (which tended to be lower after reading active embedded structures compared to the other conditions) rather point to a more systematic source of influence. First, it should be noted that adverse effects of embeddedness on oculomotor parameters in lines 1-4 were mainly present in passive conditions (see Table 2). Thus, one would expect the most pronounced effects of embeddedness on line 5 in passive conditions, which is in line with our data. If we now assume that active conditions draw (at least slightly) less cognitive resources than passive structures (e.g., due to the fact that in passive structures, additional prepositions and more words in general need to be processed), the following reasoning might be viable: In the less demanding active conditions, it is possible that the aforementioned overall disadvantage of embedded structures (greater working memory load) is compensated for by a greater ease (due to spatial proximity) of finding the anaphoric reference of the relative pronouns. Although this did not substantially affect regression rates on the first lines, it might still trigger a reduced tendency towards executing regressions when comparatively simple sentence structures are encountered (the target sentences). This would explain why slightly fewer regressions (and less second-pass processing) were observed on the target sentence after active embedded as compared to active non-embedded passages. However, since these assumptions are rather speculative, further research is certainly needed to strengthen these claims. At least, the proposed mechanism demonstrates that the somewhat deviating pattern of the late measures does not weaken the main result, which is the prolongation of first-pass reading after processing embedded structures.

It is important to note that due to the present block design, we cannot finally decide whether the adjustment of temporal oculomotor routines during first-pass reading is only due to the ease (or difficulty) of the current passage, or rather due to a sequence of easier (or more difficult) passages. However, the observation that there was no longer an effect on line 6 rather suggests an explanation in terms of a short-lasting effect, lasting for about one sentence after the processing difficulty is encountered. This assumption is also in line with previous data showing that the frequency of three consecutive words in one sentence may slow down the processing of a subsequent sentence (Slattery, Pollatsek, & Rayner, 2007), at least at the beginning of this sentence.

One might further speculate whether it is possible that expectations regarding the syntactic structure of the target sentence are generated during the reading of the first lines of each passage. Previous research on syntactic priming suggested that expectancies could indeed influence eye movements on a following target sentence, but only when the target sentence shares additional cues with the prime, for example, a repetition of specific words (e.g., Staub & Clifton, 2006). However, in priming studies, subjects usually read sentences that changed syntactic structure from sentence to sentence, and no natural text passages were used. Thus, in the present study it is more likely that the experience of overall high processing difficulty during reading embedded structures in the present passage (or, additionally, in previous passages) led to a global adjustment of eye movement patterns that maximized the time window for lexical processing, which is reflected in increased gaze durations (Rayner, 1998). Note that any differences regarding text properties in lines 1-4 across conditions that go hand in hand with the syntax manipulations do not challenge the main conclusion of the study, which is the evidence for global fine-tuning of first-pass oculomotor routines across sentence and line borders. It is possible that future research will demonstrate even more pronounced oculomotor tuning by using more effective context manipulations, such as a variation of overall word length or frequency instead of syntax.

Based on the present results, one might speculate to what extent the observed adjustment of oculomotor control reflects a cognitive strategy or, alternatively, a rather low-level automatic oculomotor routine. A related issue was raised by Rayner and Pollatsek (1989) , who linked the notion of global factors (as opposed to direct control) to automatic control, largely independent of cognition. An informal survey at the end of the experiment revealed that none of the participants was aware of any systematic text differences across the experimental blocks, suggesting that conscious awareness only played a minor role. Probably, the decision to utilize a manipulation of syntax instead of more prominent semantic variables reduced further the possibility of noticing pronounced differences between experimental conditions. However, it should be noted that even word frequency effects, which are usually referred to as prototype examples of cognitive effects, are usually not consciously experienced by the reader, and it may well be that the observed global effect is due to the difficulty of integrating the information provided by each word in the target sentence into a less comprehensible text discourse as provided by the preceding (embedded) passage. Taken together, we can only adhere to the notion that if conscious cognitive strategies play a role, it should only be minor in comparison.

In contrast to the embeddedness manipulation, we did not find evidence for increased processing difficulty for passive as compared with active sentence structures. Comprehension was not negatively affected by passive voice constructions, and only overall reading times were slightly increased for passive sentences (and only in the *F*_1_ analysis). However, this overall pattern is in line with previous research suggesting that syntax, which appears more complex at first sight (note that passive voice generally increased sentence length and the occurrence of prepositions), does not necessarily make sentences much harder to understand (e.g., Carrithers, 1989). Since there was no indication of increased processing difficulty for passive voice sentences in the first place, it is not surprising that no corresponding global fine-tuning on the target sentence occurred.

It is important to note that the present syntax manipulations inevitably go hand in hand with changes regarding other text properties, such as text length. This makes it difficult to draw any specific theoretical conclusions regarding the mechanisms of processing difficulty with embedded structures, especially since performance was overall worst in the longest text condition (passive embedded). On the other hand, the comparisons of the local effects of the syntax manipulations (on the first four lines of each passage) might rightly serve as a basis for practical recommendations regarding text readability, since any correlates of syntactic manipulations (e.g., text length) actually define their very nature: It is impossible to change syntax without systematically changing any other properties of the text. On the basis of the present data, one can draw the conclusion that readability suffers from embedded text structures to some extent, but not generally from passive voice constructions (at least in German). Furthermore, the reported interactions between voice and embeddedness suggest that the specific combination of passive voice structures within embedded sentences is especially detrimental for reading. However, it should be noted that the overall effect sizes are comparatively small (see Table 2), and many measures of processing difficulty (such as the amount of regressions back to previously inspected text) were not significantly affected. Thus, these data cannot be regarded as an empirical underpinning of the general advice to use active voice and simple sentence structures for the sake of readability (e.g., Groeben & Christmann, 1989; Strunk & White, 1918/1999).

A further interesting observation refers to the overall regression rates in the present study, which exceeded the typically reported 10-15% for reading studies (see Rayner, 2009). However, these estimates are based on experiments utilizing the presentation of single sentences and comparatively simple verification tasks to ensure comprehension. Previous research demonstrated that passage reading (as opposed to single sentence reading) and the use of more difficult comprehension questions both substantially increase regression rates (Radach et al., 2008). This is also reflected in the present data, where regression rates on the target sentence (which was always located on a single line of text) were slightly above 15%, whereas the regression rates on the first sentences (involving line breaks) amounted to more than 20%.

In sum, we showed that syntax-related text properties outside the current perceptual span affected subsequent oculomotor parameters, likely representing a global fine-tuning mechanism of eye movement control during first-pass reading. Current models of oculomotor control in reading are predominantly built around local effects of factors determining lexical processing (e.g., Engbert, Nuthmann, Richter, & Kliegl, 2005; Reichle, Rayner, & Pollatsek, 2003), but interest regarding post-lexical and syntactic processes seems to be growing (e.g., Reichle, Warren, & McConnell, 2009). Although the relative weight of global (vs. local) influences on oculomotor control cannot be determined on the basis of the present study, it seems reasonable to include a corresponding source of variance in forthcoming modelling stages to further increase their success in explaining the spatial and temporal variability of eye movement patterns. A successful implementation of global factors into the modelling of eye movement control during reading has been recently provided. Radach et al. (2008) presented evidence for effects of the reading task (comprehension vs. word verification) and the format of reading (sentence vs. passage reading) on gaze patterns. Crucially, these influences were successfully implemented into the Glenmore model of oculomotor control in reading (Reilly & Radach, 2006). The present data may serve to contribute further to the integration of global factors into our conception of eye movement control.
